# Pharmacist-Driven Rapid Initiation of Antiretroviral Therapy Decreases Time to Viral Suppression in People With HIV

**DOI:** 10.1093/ofid/ofae237

**Published:** 2024-04-26

**Authors:** Amy L Brotherton, Ann-Marie Coroniti, Diane K Ayuninjam, Martha C Sanchez, Gregorio Benitez, Joseph M Garland

**Affiliations:** Department of Pharmacy, The Miriam Hospital Infectious Diseases and Immunology Center, Providence, Rhode Island, USA; Division of Infectious Diseases, Department of Medicine, The Miriam Hospital Infectious Diseases and Immunology Center, Providence, Rhode Island, USA; Division of Infectious Diseases, Department of Medicine, Warren Alpert Medical School, Brown University, Providence, Rhode Island, USA; Department of Pharmacy, Healthcare Associates, Beth Israel Deaconess Medical Center, Boston, Massachusetts, USA; Department of Pharmacy, The Miriam Hospital Infectious Diseases and Immunology Center, Providence, Rhode Island, USA; Division of Infectious Diseases, Department of Medicine, The Miriam Hospital Infectious Diseases and Immunology Center, Providence, Rhode Island, USA; Division of Infectious Diseases, Department of Medicine, The Miriam Hospital Infectious Diseases and Immunology Center, Providence, Rhode Island, USA; Division of Infectious Diseases, Department of Medicine, Warren Alpert Medical School, Brown University, Providence, Rhode Island, USA; Division of Infectious Diseases, Department of Medicine, The Miriam Hospital Infectious Diseases and Immunology Center, Providence, Rhode Island, USA; Division of Infectious Diseases, Department of Medicine, Warren Alpert Medical School, Brown University, Providence, Rhode Island, USA; Division of Infectious Diseases, Department of Medicine, The Miriam Hospital Infectious Diseases and Immunology Center, Providence, Rhode Island, USA; Division of Infectious Diseases, Department of Medicine, Warren Alpert Medical School, Brown University, Providence, Rhode Island, USA

**Keywords:** antiretroviral therapy, HIV, pharmacist, rapid ART, viral suppression

## Abstract

**Background:**

Rapid initiation of antiretroviral therapy (rapid ART) improves clinical outcomes in people with HIV and is endorsed by clinical guidelines. However, logistical challenges limit widespread implementation. We describe an innovative rapid ART model led by pharmacists and its impact on clinical outcomes, including time to viral suppression (TVS).

**Methods:**

On 1 January 2019, we implemented Pharmacist-Driven Rapid ART (PHARM-D RAPID ART), including rapid ART initiation by pharmacists. Our retrospective cohort study compared TVS, using a Cox proportional hazards model, and clinical outcomes among individuals with a new HIV diagnosis before (1 January 2017 to 31 December 2017) and after (1 January 2019 to 31 December 2019) implementation.

**Results:**

A total of 108 individuals were included. TVS was significantly shorter (*P* < .001) for the PHARM-D RAPID ART group (n = 51) compared with the preimplementation group (n = 57) (median: 30 days and 66 days, respectively). Those in the PHARM-D RAPID ART group were significantly more likely to achieve VS at any given time during the study period (adjusted hazard ratio: 3.47 [95% confidence interval, 2.25–5.33]). A total of 94.1% (48/51) of patients in the PHARM-D RAPID ART group were retained in care at 1 year. With a median follow-up of 2.4 years in the PHARM-D RAPID ART group, 98% remained suppressed at last recorded viral load.

**Conclusions:**

A pharmacist-driven model for rapid ART delivery decreases TVS with high rates of retention in care and durable VS. This model could improve clinical outcomes and increase program feasibility and sustainability.

## Teaser

A pharmacist-driven model for rapid initiation of antiretroviral therapy in people newly diagnosed with HIV is successful, sustainable, and effective at reducing time to viral suppression.

Rapid antiretroviral (ART) programs have emerged as an innovative approach to address the well-documented attrition seen among persons newly diagnosed with human immunodeficiency virus (HIV) before ART initiation [1, 2]. In addition to improving linkage to care and time to ART initiation (TAI) for people with HIV (PWH), rapid ART programs could have significant effects on controlling the HIV epidemic by reducing the time to viral suppression (TVS) and therefore HIV transmissibility; once patients become undetectable, undetectable means untransmittable (U = U) [3]. With the current availability of newer antiretroviral agents that are highly effective, well tolerated, and simple to administer, the feasibility of a rapid-start approach has markedly increased.

Multiple studies have demonstrated that rapid ART initiation significantly reduces the rate of loss to follow-up and improves clinical outcomes among PWH during their initial engagement in HIV care [4]. Randomized controlled trials in resource-limited settings across the globe, including South Africa and Haiti, paved the way for rapid ART initiation by demonstrating increased uptake of ART, improved VS, and increased retention in care compared to standard care protocols [5–7]. Observational studies in the United States have also reported successful implementation of rapid ART programs with similar improvements in clinical outcomes and patient satisfaction [8–11]. As a result, major HIV clinical practice guidelines now recommend offering ART to any PWH interested in initiating treatment immediately on diagnosis [12–14].

Despite these recommendations and the many advantages of the rapid ART strategy, several challenges arise while implementing this protocol within a clinical setting. Rapid ART is a resource-intensive process that requires consolidation of new patient services, such as clinical evaluation, laboratory testing, patient education, addressing barriers to medication access and adherence, linkage to community services, and provision of ART, into a single visit, and clinician availability in the moment can be a significant barrier. Given that clinical pharmacists are highly accessible in a wide range of clinical settings, are experts in drug therapy, and can facilitate access to ART, shifting to a model in which pharmacists are at the forefront of the ART initiation process may provide a practical solution to the logistical challenges noted by previous studies, such as provider overburdening, clinic capacity, and barriers to medication access and acquisition [15]. Furthermore, pharmacists can leverage their clinical expertise to enhance the quality of rapid ART programs by ensuring safe and effective use of ART to reduce treatment failures and improve long-term outcomes.

Although there have been numerous studies evaluating pharmacist-led adherence interventions in PWH, there are no reports of pharmacist-driven models in the context of rapid ART in the literature [16–19]. To increase widespread implementation of rapid ART programs, it is crucial to identify innovative models that overcome barriers to successful implementation. In 2019, The Miriam Hospital Infectious Diseases and Immunology Center (TMH ID/Immunology Center) in Providence, Rhode Island, designed and implemented its pharmacist-driven rapid ART model. By comparing clinical outcomes before and after the establishment of pharmacists as the foundation of our rapid ART protocol, we report the feasibility and efficacy of this innovative model for rapid ART delivery.

## METHODS

### Study Design and Population

We conducted a single-center, quasi-experimental retrospective cohort study at TMH ID/Immunology Center, Rhode Island's largest Ryan White–funded HIV clinic that currently serves >2000 PWH. Patient demographics, socioeconomic data, and clinical data were obtained from the electronic medical record. Our study analyzed ART-naïve individuals ≥18 years of age enrolling into care with a new HIV diagnosis before and after implementation of a Pharmacist-Driven Rapid ART (PHARM-D RAPID ART) protocol. The preintervention period included individuals presenting with a new HIV diagnosis between 1 January 2017 and 31 December 2017; the postintervention period included individuals presenting with a new HIV diagnosis between 1 January 2019 and 31 December 2019. Those who were incarcerated, pregnant, or ART-experienced were also offered rapid ART if appropriate but were excluded from our study. Those enrolled in a clinical trial for ART initiation, those with a viral load of <200 copies/mL at baseline, those who declined ART initiation, or those with a contraindication to ART initiation were also excluded. Clinical contraindications to ART initiation included: suspicion for or confirmed tuberculosis meningitis, cryptococcal meningitis, or cytomegalovirus retinitis. Clinical outcomes data were collected for up to 5 years after enrollment. A waiver of consent was granted by the Lifespan institutional review board given the retrospective nature of the study.

### Preimplementation: Description of New Patient Intake Process at TMH ID/Immunology Center

Before implementation of the PHARM-D RAPID ART protocol, patients were referred to the clinic on HIV diagnosis and attended an intake visit with a nurse, which preceded their first provider appointment. Nursing evaluation included collecting patient sociodemographic information, completing clinic enrollment forms, and providing disease state education. Evaluation by a social worker for psychosocial assessment, referrals and linkage to community services, and assistance with medication coverage was conducted based on an individual patient's needs. Patients were not evaluated by a pharmacist, and ART initiation was deferred to provider visit. ART access issues were resolved when reported by the patient or the dispensing pharmacy. There was no model for rapid ART before PHARM-D RAPID ART implementation.

The first clinic-based pharmacist was hired in September 2017. From 1 January 2018 to 31 December 2018, pharmacists were consulted on a case-by-case basis to provide patient counseling during the intake visit or resolve medication access issues to decrease barriers to ART initiation. In limited circumstances, patients were offered rapid ART. Because of the mixed model, data from 2018 were not included in the analysis to provide a more direct comparison of the program with the prior intake protocol.

### Intervention: PHARM-D RAPID ART Model

**Figure 1.**
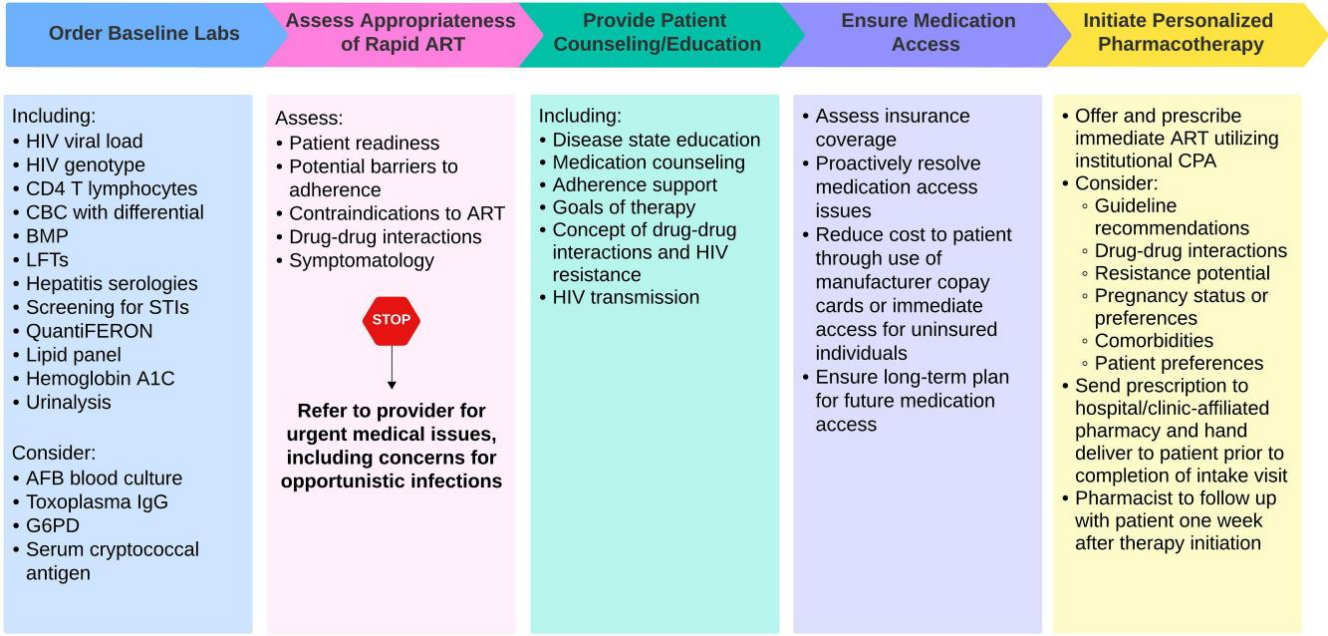
Role of the pharmacist in the PHARM-D RAPID ART model. Abbreviations: AFB, acid-fast bacilli; ART, antiretroviral therapy; BMP, basic metabolic panel; CBC, complete blood count; CPA, collaborative practice agreement; G6PD, glucose-6-phosphate-dehydrogenase; HIV, human immunodeficiency virus; IgG, immunoglobulin G; LFTs, liver function tests; STIs, sexually transmitted infections.

Beginning 1 January 2019, we modified our new patient intake process to allow for a multidisciplinary visit, including clinical evaluation and same-day ART initiation by pharmacists before a patient's first provider visit. After initial nursing evaluation, clinical pharmacists followed the algorithm and tasks outlined in Figure 1, supported through a Collaborative Practice Agreement (CPA). When insurance allowed, prescriptions were filled through our hospital-affiliated pharmacy and hand delivered to the patient before completion of the visit. Evaluation by a social worker was conducted based on an individual patient's needs. Pharmacists conducted follow-up telephone calls 1 week after ART initiation for continued counseling, adherence support, and assessment of medication tolerability. Further visits occurred as needed thereafter until the patient had achieved an undetectable viral load and attended 2 provider visits. At the time of the intervention, the clinic had 2 dedicated pharmacists with specialized training and credentials in HIV pharmacotherapy and infectious diseases.

### Definitions and Outcomes

Retention in care was defined as 2 provider visits separated by at least 90 days within 1 year from date of intake. In the preimplementation group, TAI was defined as date of provider prescribing ART in the electronic medical record because date of first dose of ART could not be confirmed. In the PHARM-D RAPID ART group, TAI was defined as time to first dose administered as reported in the electronic medical record. Number of encounters with the healthcare team included both provider visits (defined as physician or advance practice provider [APP] visits, urgent care visits, emergency department visits, or hospitalizations); and nonbillable encounters (eg, visits or phone calls with clinic staff members, including pharmacists, nurses, or social workers).

The primary outcome compared time from clinic intake visit to VS (ie, HIV RNA <200 copies/mL) at 1 year between the 2 groups. We also conducted a sensitivity analysis in which we compared time from positive confirmatory HIV testing results to viral suppression at 1 year between the 2 groups.

Secondary outcomes included (1) time from intake visit to first provider visit, (2) time from intake visit to AI, (3) retention in care at 1 year from intake visit, (4) VS rates at 1 year from intake visit, (5) VS rates at last recorded viral load, (6) adverse events leading to increased monitoring or discontinuation, and (7) number of encounters with the healthcare team within 90 days of intake date. Additionally, we descriptively report the total length of follow-up time for both groups.

### Statistical Analysis

Continuous variables were presented as medians with either 95% confidence intervals (CI) or interquartile ranges (IQR). Univariate analyses between groups were compared using Wilcoxon rank-sum test for continuous variables and either Pearson chi-square test or Fisher exact test, as appropriate, for categorical variables.

For both the primary analysis and sensitivity analysis, unadjusted Kaplan-Meier survival curves were constructed to depict cumulative viral suppression over the 1-year period, and group comparisons were made using log-rank tests. Specifically for the primary analysis, Cox proportional hazards models were used to estimate time to viral suppression by intervention group. In the adjusted Cox model, time to viral suppression was adjusted for baseline HIV RNA level (ie, ≤100 000 copies/mL or >100 000 copies/mL) and presence of either CD4 cell count <200 cells/mm^3^ or an AIDS defining illness. Patients were right-censored if they did not reach viral suppression by the end of the study period, and the proportional hazards assumption was checked with the Schoenfeld global test.

A *P* value of <.05 was considered statistically significant. For our analyses, we only included individuals without any missing data. Analyses were performed using R software (version 4.2.2).

## RESULTS

### Study Disposition

**Figure 2.**
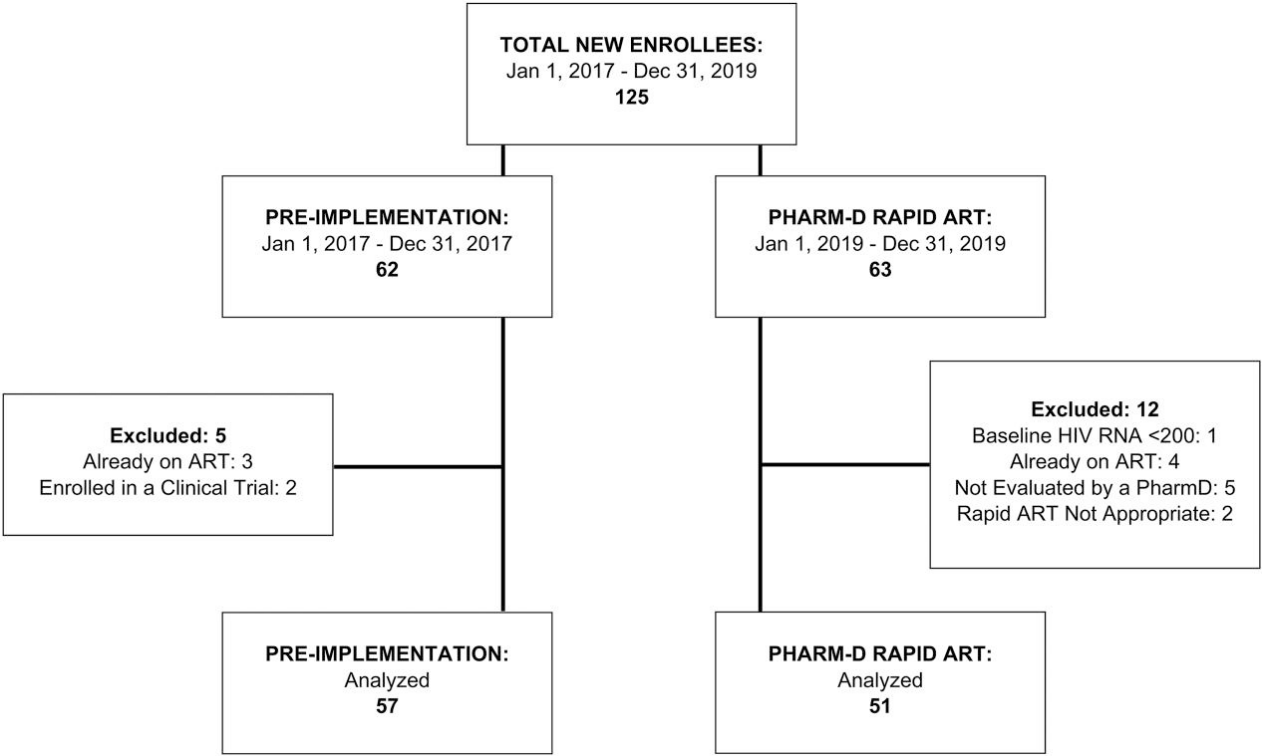
Study enrollment flow chart.

A total of 125 patients newly diagnosed with HIV were evaluated for inclusion in the study. A total of 108 patients were included in the final analysis: 57 in the preimplementation group and 51 in the PHARM-D RAPID ART group. Seventeen patients (5 in the preimplementation group, 12 in the PHARM-D RAPID ART group) were excluded from the analysis for the following reasons: HIV RNA <200 copies/mL at baseline (n = 1), already on ART (n = 7), not evaluated by a pharmacist (n = 5), rapid ART was deemed not appropriate (n = 2), or enrolled in a clinical trial (n = 2) (Figure 2). Only 1 patient declined rapid initiation of ART by a pharmacist. No patients died during the study period.

### Baseline Characteristics

Baseline sociodemographic and clinical characteristics were similar between the 2 groups (Table 1). Median age for the entire cohort was 35 (IQR 27–47). Most patients identified as male (92/108, 85.2%) and as men who have sex with men (59/108, 54.6%). Half of patients (56/108, 51.8%) disclosed having current or previous substance use disorder or mental health disorder, 27.8% (30/108) of patients were uninsured, and 14.8% (16/108) of patients reported unstable housing. Median CD4 cell count at diagnosis for the entire cohort was 451 cells/mm^3^ (IQR 239–582 cells/mm^3^). Most patients (106/108, 98%) were initiated on an integrase strand transfer inhibitor–based regimen in combination with dual nucleoside reverse transcriptase inhibitors, commonly elvitegravir or dolutegravir in the preimplementation group and bictegravir in the PHARM-D RAPID ART group, reflective of guideline recommendations at the time. Of note, 3 patients in the PHARM-D RAPID ART group were initiated on an elvitegravir-based regimen because of payer restrictions. Median time from confirmatory testing to intake visit for the entire cohort was 5 days (IQR 1–9 days). Patients in the PHARM-D RAPID ART group initiated ART a median of 6 days from date of positive confirmatory testing (IQR 1–9 days). Clinically significant baseline HIV resistance was present in 21.3% (23/108) of patients, mostly commonly nonnucleoside reverse transcriptase inhibitor resistance. An indication for prophylaxis or treatment of opportunistic infections was present in 22.2% (24/108) of patients. There were no cases where rapid ART was deemed clinically inappropriate because of presence of or suspicion for central nervous system opportunistic infections.

**Table 1.** Sociodemographic and Clinical Characteristics of Participants at Baseline

		Pharm-D Rapid ART N = 51		
Characteristic	Preimplementation N = 57	No. (%) or Median (IQR)	Total N = 108	*P* Value
Age	37 (27–48)	31 (27–46)	35 (27–47)	.50
Sex assigned at birth				.589
Female	7 (12.3%)	9 (17.6%)	16 (14.8%)	
Male	50 (87.7%)	42 (82.4%)	92 (85.2%)	
Race/ethnicity				.627
Hispanic	19 (33.3%)	12 (23.5%)	31 (28.7%)	
Non-Hispanic Black	10 (17.5%)	13 (25.5%)	23 (21.3%)	
Non-Hispanic White	25 (43.9%)	24 (47.1%)	49 (45.4%)	
Other	3 (5.3%)	2 (3.9%)	5 (4.6%)	
Language				.668
English	46 (80.7%)	41 (80.4%)	87 (80.6%)	
Spanish	7 (12.3%)	5 (9.8%)	12 (11.1%)	
Cape Verdean Creole	2 (3.5%)	4 (7.8%)	6 (5.6%)	
Haitian Creole	1 (1.8%)	0 (0%)	1 (0.93%)	
Portuguese	0 (0%)	1 (2.0%)	1 (0.93%)	
ASL	1 (1.8%)	0 (0%)	1 (0.93%)	
Risk factor for acquisition				.964
Heterosexual	16 (28.1%)	13 (25.5%)	29 (26.9%)	
IDU	1 (1.8%)	0 (0%)	1 (0.9%)	
MSM	31 (54.4%)	28 (54.9%)	59 (54.6%)	
Multiple risk factors	9 (15.8%)	10 (19.6%)	19 (17.6%)	
Insurance				.14
Uninsured	19 (33.3%)	11 (21.6%)	30 (27.8%)	
Medicaid	21 (36.9%)	17 (33.3%)	38 (35.2%)	
Medicare	0 (0%)	1 (2.0%)	1 (0.9%)	
Private	17 (29.8%)	22 (43.1%)	39 (36.1%)	
Unstable housing				1.00
No	8 (14.0%)	8 (15.7%)	16 (14.8%)	
Yes	49 (86.0%)	43 (84.3%)	92 (85.2%)	
Annual income, USD	7200 (0–26 000)	18 000 (0–32 000)	11 880 (0–29 060)	.451
Education				.759
Less than HS	10 (17.5%)	12 (23.5%)	22 (20.4%)	
HS	15 (26.3%)	12 (23.5%)	27 (25.0%)	
Beyond HS	32 (56.1%)	27 (52.9%)	59 (54.6%)	
Mental health diagnosis				.26
No	31 (54.4%)	21 (41.2%)	52 (48.1%)	
Current	18 (31.6%)	24 (47.1%)	42 (38.9%)	
Past	8 (14.0%)	6 (11.8%)	14 (13.0%)	
Substance use				.26
No	31 (54.4%)	21 (41.2%)	52 (48.1%)	
Active	18 (31.6%)	24 (47.1%)	42 (38.9%)	
Past	8 (14.0%)	6 (11.8%)	14 (13.0%)	
CD4 cell count <200 or AIDS defining illness	15 (26.3%)	9 (17.6%)	24 (22.2%)	.356
Absolute CD4 cell count (cells/μl)	424.0 (191.0–552.0)	491.5 (355.5–601.3)	451.0 (239.0–582.0)	.113
HIV-1 RNA > 100 000 copies/mL	24 (42.1%)	13 (25.5%)	37 (34.3%)	.104
Log_10_ HIV-1 RNA	4.76 (4.16–5.48)	4.55 (3.96–5.09)	4.70 (4.13–5.21)	.0518
Time from confirmatory testing positive to intake date, d	4.00 (0.00–11.00)	6.00 (1.00–9.00)	5.00 (1.00–9.50)	.357
Presence of STIs at time of diagnosis	18 (31.6%)	19 (37.3%)	37 (34.3%)	.550
HBV coinfection	2 (3.5%)	3 (5.9%)	5 (4.6%)	.665
HCV coinfection	1 (1.8%)	1 (2.0%)	2 (1.9%)	1.00
History of PrEP use	1 (1.8%)	3 (5.9%)	4 (3.7%)	.342
Medication started				<.001
EFV + ABC/3TC	1 (1.8%)	0 (0%)	1 (0.9%)	
EVG/c/TAF/FTC	42 (73.7%)	1 (2.0%)	43 (39.8%)	
RPV/TAF/FTC	1 (1.8%)	0 (0%)	1 (0.9%)	
EVG/c/TDF/FTC	3 (5.3%)	2 (3.9%)	5 (4.6%)	
DTG + TAF/FTC	2 (3.5%)	0 (0%)	2 (1.9%)	
DTG + TDF/FTC	1 (1.8%)	2 (3.9%)	3 (2.8%)	
DTG/ABC/3TC	7 (12.3%)	0 (0%)	7 (6.5%)	
BIC/TAF/FTC	0 (0%)	46 (90.2%)	46 (42.6%)	
Clinically significant baseline HIV resistance				.794
NNRTI	10 (17.5%)	10 (19.6%)	20 (18.5%)	
2+ classes	1 (1.8%)	2 (3.9%)	3 (2.8%)	
Unknown	4 (7.0%)	2 (3.9%)	6 (5.6%)	
OI prophylaxis or treatment indicated	15 (26.3%)	9 (17.6%)	24 (22.2%)	.358

Univariate analyses between groups were compared using Wilcoxon rank-sum test for continuous variables and either Pearson chi-square test or Fisher exact test, as appropriate, for categorical variables.

Abbreviations: 3TC, lamivudine; ABC, abacavir; ASL, American Sign Language; BIC, bictegravir; DTG, dolutegravir; EFV, efavirenz; EVG/c, elvitegravir/cobicistat; FTC, emtricitabine; HBV, hepatitis B virus; HCV, hepatitis C virus; HS, high school; IDU, injection drug use; IQR, interquartile range; MSM, men who have sex with men; NNRTI: nonnucleoside reverse transcriptase inhibitor; OI, opportunistic infection; PrEP, preexposure prophylaxis; RPV, rilpivirine; STI, sexually transmitted infections; TAF, tenofovir alafenamide; TDF, tenofovir disoproxil fumarate; USD, US dollar.

### Time to Viral Suppression

**Figure 3.**
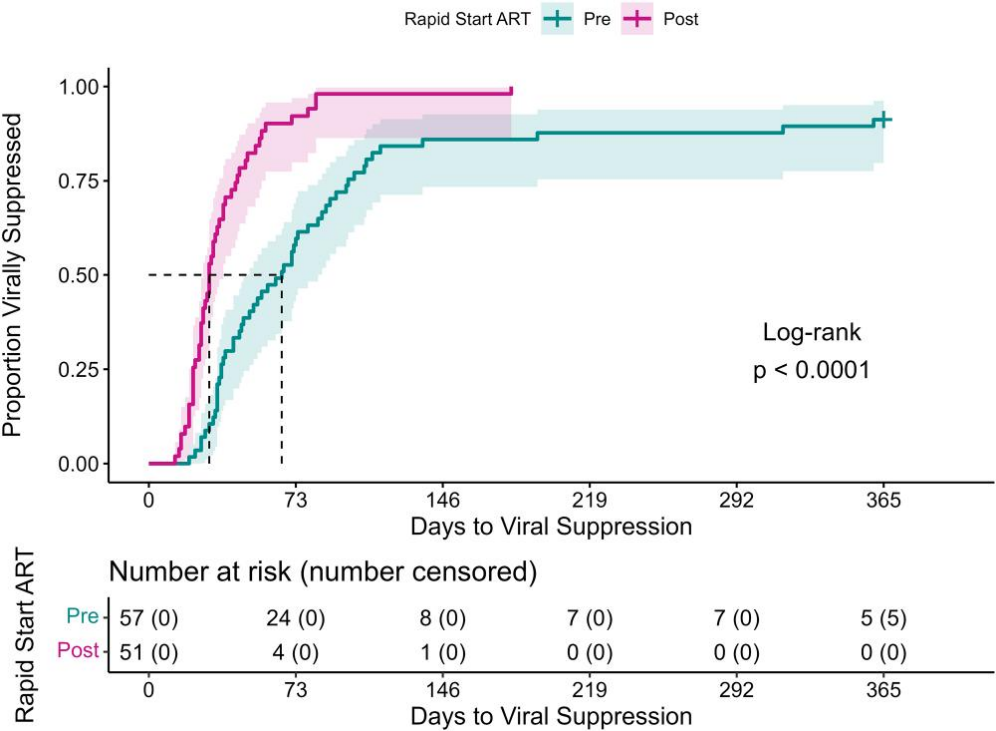
Primary outcome: time to viral suppression, time (days) to viral suppression (ie, HIV RNA < 200 copies/mL) up to 365 d following presentation to the Miriam Hospital Infectious Diseases & Immunology Center for intake (day 0). The unadjusted Kaplan-Meier curves show the proportion of patients who reached viral suppression over time from either the cohort that followed the pharmacist-driven rapid initiation of ART protocol (Post-Rapid Start ART) or the cohort of historical controls (Pre-Rapid Start ART). Compared with the Pre-Rapid Start ART cohort (median 66 d), time to viral suppression among the Post-Rapid Start ART cohort (median 30 d) was significantly shorter (*P* < .0001), as evaluated by the log-rank test. Five patients in the Pre-Rapid Start ART cohort did not reach suppression by the end of the study period. Abbreviation: ART, antiretroviral therapy.

TVS was significantly shorter (*P* < .001) for the PHARM-D RAPID ART group compared with the preimplementation group (median: 30 days [95% CI, 27–37] and 66 days [95% CI, 47–84], respectively) (Figure 3). The unadjusted hazard ratio for VS was 3.28 (95% CI, 2.16–4.99), whereas the adjusted hazard ratio for VS was 3.47 (95% CI, 2.25–5.33). The proportional hazard assumption was not violated (global test: *P* = .14).

**Figure 4.**
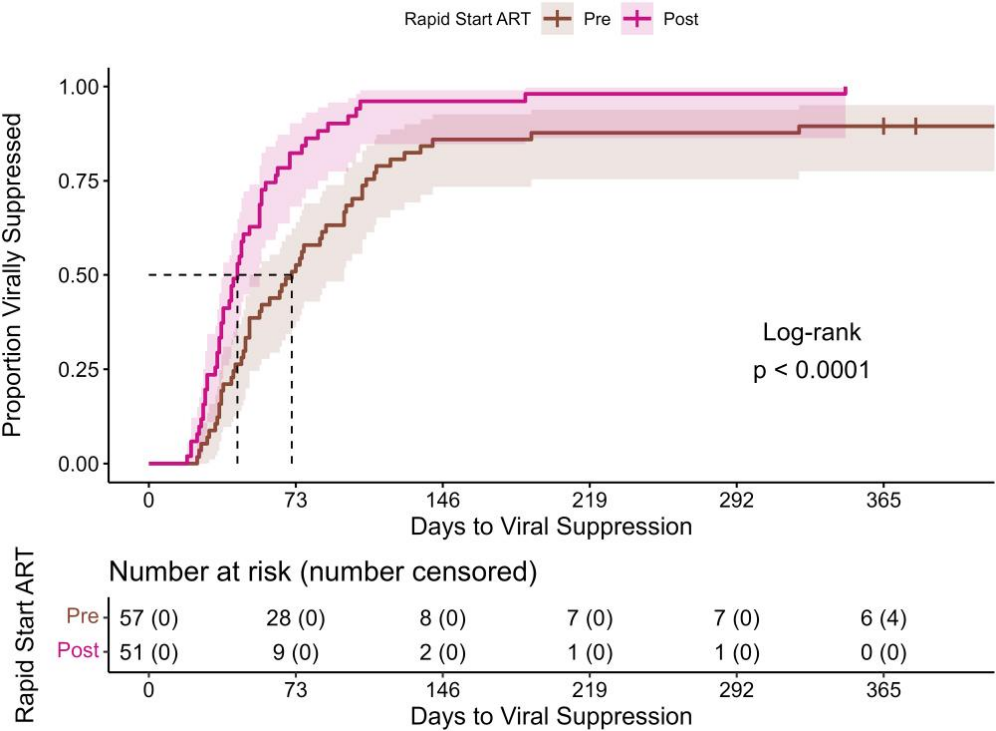
Time from confirmatory testing to viral suppression, time (days) to viral suppression (ie, HIV RNA < 200 copies/mL) up to 365 d following confirmatory testing at the Miriam Hospital Infectious Diseases & Immunology Center (day 0). The unadjusted Kaplan-Meier curves show the proportion of patients who reached viral suppression over time from either the cohort that followed the pharmacist-driven rapid initiation of ART protocol (Post-Rapid Start ART) or the cohort of historical controls (Pre-Rapid Start ART). Compared with the Pre-Rapid Start ART cohort (median 71 d), time to viral suppression among the Post-Rapid Start ART cohort (median 44 d) was significantly shorter (*P* < .0001), as evaluated by the log-rank test. Four patients in the Pre-Rapid Start ART cohort did not reach suppression by the end of the study period. Abbreviation: ART, antiretroviral therapy.

In our sensitivity analysis, time from positive confirmatory testing to VS was also significantly shorter (*P* < .001) for the PHARM-D RAPID ART group compared with the preimplementation group (median 44 days [95% CI, 50–97] and 71 days [95% CI, 50–97], respectively) (Figure 4).

### Secondary Analyses

Time from intake visit to provider visit was significantly longer (*P* < .019) for the PHARM-D RAPID ART group compared with the preimplementation group (median 20 days [IQR: 10.50–27.50] and 9 days [IQR: 3–20], respectively), whereas TAI was significantly shorter (*P* < .001) in the PHARM-D RAPID ART group compared with the preimplementation group (median 0 days [IQR: 0–0] and 9 days [IQR: 3–20], respectively) (Table 2). Additionally, the total number of encounters with the health care team within 90 days from intake were greater (*P* < .001) for the PHARM-D RAPID ART group compared with the preimplementation group (median 15 encounters [IQR: 12–19.50] and 9 encounters [IQR: 8–12], respectively). Of note, the median number of physician/APP clinic visits, urgent care visits, emergency department visits, or hospitalizations within 90 days from intake were 2 (IQR: 1–3) and 4 (IQR: 3–4), for the PHARM-D RAPID ART group compared with the preimplementation group, respectively.

**Table 2.** Comparison of Clinical and Process Outcomes for Patients Newly Diagnosed With HIV at TMH ID/Immunology Center

	Preimplementation N = 57	Pharm-D Rapid ART N = 51	*P* Value
Variable	No. (%) or Median (IQR)		
Time to MD visit from intake, d	9 (3–20)	20 (10.50–27.50)	.019
Time to ART initiation from intake, ds	9 (3–20)	0 (0–0)	<.001
Virologically suppressed at last recorded viral load	52 (91.2%)	50 (98.0%)	.210
Not virologically suppressed at 1 y from intake	5 (8.8%)	0 (0%)	.590
Retention in care at 1 year from intake	49 (86.0%)	48 (94.1%)	.210
Adverse effects leading to increased monitoring	2 (3.5%)	1 (2.0%)	1.00
Regimen changed because of baseline resistance	1 (1.8%)	2 (3.9%)	.601
Number of encounters with health care team within 90 d from intake			<.001
Total	9 (8–12)	15 (12–19.50)	
Provider encounters	4 (3–4)	2 (1–3)	

Univariate analyses between groups were compared using Wilcoxon rank-sum test for continuous variables and either Pearson chi-square test or Fisher exact test, as appropriate, for categorical variables. Provider encounters were defined as physician or advance practice provider clinic visits, urgent care visits, emergency department visits, or hospitalizations.

Abbreviations: ART, antiretroviral therapy; IQR, interquartile range; MD, medical doctor.

There were no significant differences in rates of retention in care at 1 year (94.1% vs 86%, *P* = .210), VS at any time during 1-year follow-up period (100% vs 91.2%, *P* = .590), and VS at last recorded viral load (98% vs 91.2%, *P* = .210) in the PHARM-D RAPID ART group compared with the preimplementation group, respectively.

Mild to moderate adverse effects occurred in 21% (23/108) of all patients, most commonly gastrointestinal upset (16/23, 69.6%). Adverse effects requiring increased monitoring or regimen discontinuation were rare in both groups, and only 1 patient, from the PHARM-D RAPID ART group, required modification of ART because of an adverse effect, which was triaged by the pharmacist.

Descriptively, median follow-up time was 2.40 years [IQR: 1.43–2.75] and 4.45 years [IQR: 4.12–4.74] in the PHARM-D RAPID ART group and preimplementation group, respectively.

## DISCUSSION

This report describes the successful implementation of an innovative rapid ART program in an urban HIV clinic in the United States. The program used clinic-based pharmacists to provide immediate access to ART for individuals newly diagnosed with HIV, introducing a new model for rapid HIV care. Results demonstrated that both the TAI and TVS were significantly reduced with the PHARM-D RAPID ART model. At any time point during the study period from entry into care, nearly 4 times as many patients in the PHARM-D RAPID ART group had achieved VS compared with the preimplementation group. Additionally, rates of retention in care were high, and nearly all patients in the PHARM-D RAPID ART group were virologically suppressed at the last recorded viral load after 2.4 years of follow-up, which circumvented a key limitation of previous studies by demonstrating durable clinical success.

Prior studies both internationally and within the United States have demonstrated similar positive outcomes from rapid ART programs, including rapid VS and high patient satisfaction, leading to all major guidelines recommending rapid ART initiation [4–11, 12–14]. However, logistical challenges have hindered widespread adoption of this approach within the structure of US-based clinics. To facilitate broader dissemination of rapid ART programs in the United States, Koester and colleagues identified 7 key elements for successful program implementation [15]. Among these, our pharmacists played a crucial role in 4 areas: evaluating new patients and prescribing ART, expanding clinic capacity for new patient visits, facilitating immediate medication access, and enhancing the patient-centered approach and quality of patient care.

Clinical pharmacists have long served an integral role in medication management for PWH, leveraging their expertise in medication safety, tolerability, and monitoring to improve ART adherence. Consistent adherence to ART is crucial for VS, prevention of viral transmission, and reducing the risk of development of HIV resistance and advanced disease. Several studies have demonstrated that pharmacist involvement in HIV care leads to improved ART adherence and clinical outcomes, including VS rates and rise of CD4 cell count [19]. Nevo and colleagues found that clinical pharmacist-based ART initiation, through a CPA, resulted in a higher likelihood of achieving VS during the first two years of ART and longer durability of the first ART regimen [18]. Our study further supports the value of the pharmacist-driven ART model in improving patient outcomes and expands the role of pharmacists to include rapid initiation of ART.

In many clinics serving PWH, the time to a new provider appointment is often a barrier to engagement in care for new patients. Allocating same-day patient appointment slots for new diagnoses is rarely feasible or sustainable because of provider availability and limited clinic resources. To address this issue, some innovator sites have hired APPs to participate in the provision of rapid ART [15]. Clinic-based pharmacists, although not universal, are commonly employed in Ryan White–funded clinics throughout the United States, and ambulatory care pharmacists are frequently employed in both community health centers and ambulatory clinics. The flexibility of our pharmacists’ schedules to accommodate same-day patient visits expanded program capacity and improved feasibility of implementation of rapid ART in our clinic. Furthermore, this allowed us to stretch the time to first provider visit, decreasing burden on providers, while still providing patients with excellent care.

A challenge with implementing rapid ART in the United States is ensuring ART coverage, which can further delay treatment initiation. Without dedicated services to proactively address medication access, patients may not start ART in a timely manner, even when prescribed. Previous rapid ART models without pharmacist involvement used ART starter packs to provide immediate ART, but this resource is not widely available in all settings. In addition to their clinical expertise, pharmacists are knowledgeable about payer systems and funding mechanisms for immediate medication access. Clinic pharmacists can collaborate with their counterparts in community or hospital-based pharmacies to ensure successful delivery of ART. In our model, pharmacists proactively resolved barriers to access, reduced copays for patients, and ensured a long-term plan for ART coverage, contributing to sustainable medication access. Experiencing barriers at the pharmacy can be traumatizing for patients and may result in disengagement in care or decreased adherence. In our model, prescriptions were filled at our hospital-affiliated pharmacy and hand-delivered to the patient by the pharmacist on conclusion of the visit, providing patients with a seamless process for medication acquisition, and exemplifying the “red carpet” patient-centered approach that rapid ART programs aim to achieve. Through collaboration with our hospital-affiliated pharmacy, we were also able to financially justify additional pharmacist positions and other patient-directed services at our clinic.

Overall, pharmacist-driven rapid ART offers a beneficial and sustainable approach to providing access to rapid ART services. The combination of unique clinical expertise, accessibility, and ability to navigate ART access positions pharmacists as central figures in rapidly assessing new patients for ART initiation. Our PHARM-D RAPID ART model was highly acceptable and feasible to patients, providers, and clinic staff. To date, the PHARM-D RAPID ART protocol in our center has been in effect for more than 5 years. The long-term success of our program demonstrates the potential for this model to be implemented in other clinics across the United States, to overcome logistical barriers and provider burnout noted in other rapid ART studies, increase feasibility of implementation, enhance patient-centered care, and improve clinical outcomes for PWH.

### Limitations and Future Directions

There were some limitations to this study that may affect its broad applicability. Rhode Island is a low-incidence state for HIV, and therefore our sample size was limited to a total of 108 patients. Further, as a quasi-experimental retrospective cohort study, we cannot fully account for temporal differences between the study years of the intervention and the historical comparator that may have impacted access or TAI, though notably regimens and approaches were comparable between the 2 groups.

Additionally, there were certain groups with minimal or no data available because of the limited sample size. Pregnant individuals were excluded, as our clinic policy requires that all pregnant individuals see a provider at time of treatment initiation, and people who inject drugs were not well represented. The Rhode Island Department of Health reports that less than 5 new HIV diagnoses per year in the state are attributable to injection drug use; the demographic data of the patients in this study reflect that reality.

Though common in Ryan White–funded clinic settings, pharmacists are not universal to ambulatory settings. Pharmacists also fill many roles, and the role described here may not be feasible in all settings. Further, the prescribing agreement used in this study (a CPA) is state-specific, and individual programs would need to understand the possibilities open to them in different states or countries. That said, the role of pharmacists in ambulatory care has been consistently expanding, with recent laws passed in multiple states supporting pharmacist prescribing of HIV post-exposure prophylaxis and pre-exposure prophylaxis as just some of the latest examples. Our results emphasize the value of incorporating a pharmacist into the multidisciplinary HIV ambulatory care model.

Further study of this type of intervention, perhaps through a large multicenter study with long-term follow-up, might elucidate additional benefits such as potential differences in long-term retention in care, health outcomes, and barriers or supports for ongoing medication access for individuals initiated on rapid ART by a clinical pharmacist. Formal evaluation of patient satisfaction would also be warranted to help further refine program goals and structure.

Of note, there were a greater number of encounters with the health care team in the PHARM-D RAPID ART arm of this study within the first 90 days of entry to care. However, this number does not reflect a higher number of physician/APP clinic visits, urgent care visits, emergency department visits, or hospitalizations; rather, it reflects additional interactions with the pharmacists and clinical staff to address ART adherence, tolerability, and medication access, or other patient concerns. Engagement with the clinic is key to long-term retention in care, therefore we believe that this finding further supports the principal outcome.

## CONCLUSIONS

To our knowledge, this report is the first to describe a pharmacist-driven model for implementation of a rapid ART program in an urban HIV clinic. This study demonstrated success with an innovative model for rapid ART using staff that are common to many Ryan White–funded clinics and federally qualified health centers. A major perceived barrier to rapid ART rollout is the reality of tight clinician schedules and long wait times for new patient visits. This model presents a novel approach to engaging patients rapidly that is sustainable and effective at reducing viral load in individuals newly diagnosed with HIV.

## Notes

***Acknowledgments.*** All authors take responsibility for the accuracy of the discussion and had authority over manuscript preparation and the decision to submit.

The authors thank Rajeev Shah, PharmD, Brenna Reilly-Evans, PharmD, Jennie Johnson, MD, Fizza Gillani, PhD, the Rhode Island Department of Health, the staff of TMH ID/Immunology Center, and the patients of TMH ID/Immunology Center and their families.

***Patient consent statement.*** Our study does not include factors necessitating patient consent.

***Financial support.*** This research did not receive any specific grant from funding agents in the public, commercial, or not-for-profit sectors.
